# Safety of *Pseudomonas chlororaphis* as a gene source for genetically modified crops

**DOI:** 10.1007/s11248-018-0061-6

**Published:** 2018-02-09

**Authors:** Jennifer A. Anderson, Jamie Staley, Mary Challender, Jamie Heuton

**Affiliations:** DuPont Pioneer, 8325 NW 62nd Avenue, Johnston, IA 50131 USA

**Keywords:** Genetically modified crops, *Pseudomonas chlororaphis*, Safety assessment, Insect protection, Agricultural biotechnology

## Abstract

Genetically modified crops undergo extensive evaluation to characterize their food, feed and environmental safety prior to commercial introduction, using a well-established, science-based assessment framework. One component of the safety assessment includes an evaluation of each introduced trait, including its source organism, for potential adverse pathogenic, toxic and allergenic effects. Several *Pseudomonas* species have a history of safe use in agriculture and certain species represent a source of genes with insecticidal properties. The *ipd072Aa* gene from *P. chlororaphis* encodes the IPD072Aa protein, which confers protection against certain coleopteran pests when expressed in maize plants. *P. chlororaphis* is ubiquitous in the environment, lacks known toxic or allergenic properties, and has a history of safe use in agriculture and in food and feed crops. This information supports, in part, the safety assessment of potential traits, such as IPD072Aa, that are derived from this source organism.

## Introduction

Genetically modified (GM) crops were first commercialized in the mid-1990s and currently are planted on over 90% of corn, cotton and soybean acres in the United States (USDA-NASS 2017). GM crop adoption continues to increase globally, due to their economic and sustainability benefits (Anderson et al. 2016; ISAAA 2016). Most commercial GM crops containing insect protection traits currently rely on genes derived from *Bacillus thuringiensis* (*Bt*) to provide selective protection against economically important pests. The safety of *Bt* as a source of insecticidal genes for GM crops is well established (Box 1). *Bt* was initially developed as a microbial pesticide spray and has a history of safe use in agriculture when applied, as intended, on food and feed crops (US-EPA 1998). *Bt* is ubiquitous in the environment (Schnepf et al. 1998), non-toxic to mammals and does not have pathogenic or allergenic properties (US-EPA 1998).

Pseudomonads are rod-shaped, aerobic, gram-negative bacteria. Certain *Pseudomonas* species have previously been reported to have entomopathogenic properties and represent a promising source of insecticidal genes for use in GM crops (Kupferschmied et al. 2013). A gene, *ipd072Aa*, from *Pseudomonas chlororaphis*, which encodes the IPD072Aa protein, has recently been reported to confer protection against certain coleopteran pests when expressed in maize plants (Schellenberger et al. 2016).

The safety assessment framework for GM crops is well established and has been adopted globally to evaluate a variety of trait types, including those for insect protection (Codex Alimentarius Commission 2009; EFSA 2006; FAO/WHO 1991). The assessment includes, in part, an evaluation of each introduced trait, including its source organism, for potential adverse pathogenic, toxic and allergenic effects (Delaney et al. 2008). This paper provides an assessment of the safety of *P*. *chlororaphis* as a gene source for GM crops. Like *Bt*, certain species of *Pseudomonas* including *P*. *chlororaphis* are ubiquitous in the environment, have a history of safe use in agriculture as seed treatments, foliar-applied biopesticides and as a gene source for GM crops, and lack known pathogenic, toxic or allergenic properties. This information supports, in part, the safety assessment of potential traits, such as IPD072Aa, derived from this source organism.

### Ubiquity in the environment

**Fig. 1:**
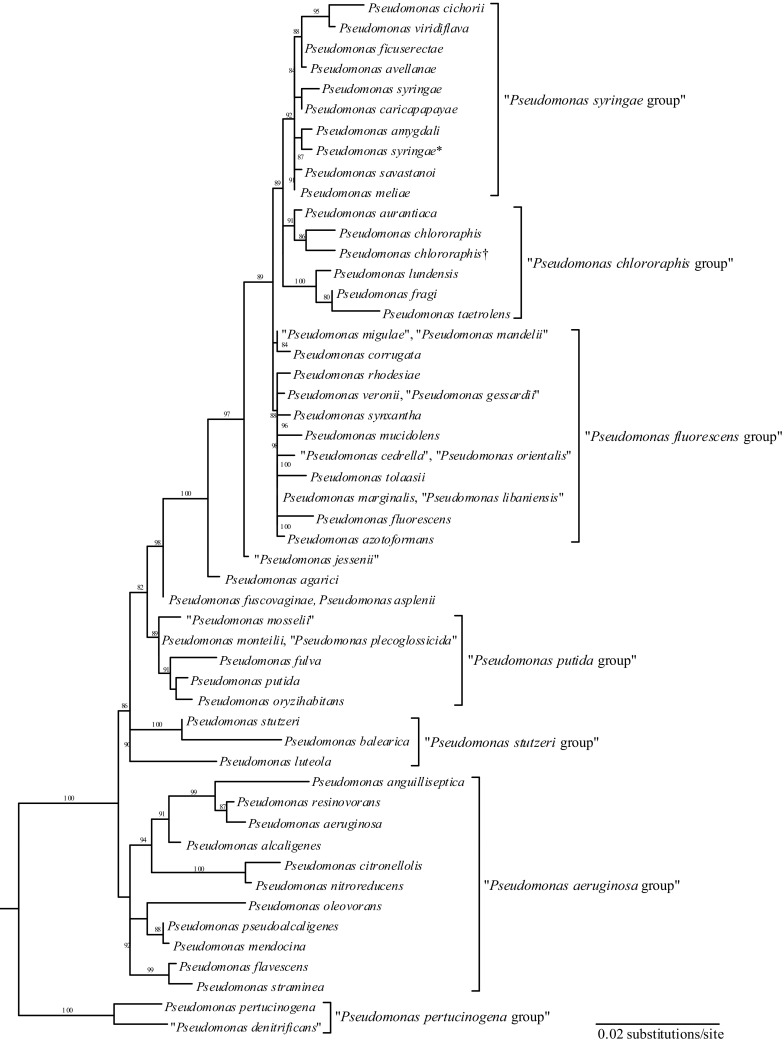
Phylogenetic tree of the authentic *Pseudomonas* derived from the similarities of the 16S rDNA sequence. Bootstrap percentages of 80% or more are indicated at the branch points. *Escherichia coli* (V00348) is used as the root organism. Symbols: *previously *P. coronafaciens*; ^†^previously *P. aureofaciens*. Reproduced with permission from Anzai et al. 2000, © International Union of Microbiological Sciences

The genus *Pseudomonas* has been well studied and is estimated to contain over 100 species and 10 sub-species (Gomila et al. 2015; Peix et al. 2009). Sequence analysis of conserved housekeeping genes has provided information on the phylogenetic relatedness of *Pseudomonas* species within the genus (Anzai et al. 2000; Garrity et al. 2005; Gomila et al. 2015; Moore et al. 2006). *Pseudomonas* species have been classified into 7 groups: *P*. *syringae*, *P*. *chlororaphis*, *P*. *fluorescens*, *P*. *putida*, *P*. *stutzeri*, *P*. *aeruginosa* and *P*. *pertucinogena* (Anzai et al. 2000; Fig. 1). *P*. *chlororaphis* contains four subspecies: *P*. *chlororaphis* subsp. *aurantiaca*, *P*. *chlororaphis* subsp. *aureofaciens*, *P*. *chlororaphis* subsp. *chlororaphis* and *P*. *chlororaphis* subsp. *piscium* (Burr et al. 2010).

Most *Pseudomonas* species, including *P*. *chlororaphis*, are ubiquitous in the environment, have widespread distribution in soil and water (Peix et al. 2009) and perform a range of economic services and ecological functions. Some *Pseudomonas* species inhabit the rhizosphere, are associated with plant roots and provide benefits to the plant by competing with soil-borne plant pathogens and protecting against fungal pests (Anderson and Kim 2018; Kupferschmied et al. 2013; Mauchline and Malone 2017). *P*. *chlororaphis*, specifically, has been reported to promote plant growth, stimulate microbial communities and protect plants by producing compounds (e.g., phenazine-type antibiotics, hydrogen cyanide, chitinases and proteases) that inhibit fungal growth (EFSA 2015b), insects and nematodes (Anderson and Kim 2018). Other *Pseudomonas* species protect plants by preventing colonization by deleterious microorganisms (Mendes et al. 2011).

Certain *Pseudomonas* species have been utilized in a variety of applications, including the biological control of phytopathogens (Walsh et al. 2001), promotion of plant growth (Mercado-Blanco and Bakker 2007), phosphate solubilization (Rodríguez and Fraga 1999) and bioremediation of organic compounds (Moore et al. 2006; Peix et al. 2009). Many *Pseudomonas* species have a history of safe use in agriculture and other sectors (EFSA 2015b; Montie 1998). For example, certain *Pseudomonas* species are entomopathogenic and are being utilized as biopesticides to provide plant protection against insect pests. Insecticidal toxins in the genome of *P*. *entomophilia* have been identified (Luiu et al. 2013), and *P*. *fluorescens* has been shown to exert insecticidal activity against aphids, termites and other agricultural pests (Kupferschmied et al. 2013). Similarly, other species of *Pseudomonas*, including *P*. *chlororaphis*, *P*. *protegens* and *P*. *aeruginosa*, have demonstrated insecticidal activity (see Table 2 of Kupferschmied et al. 2013). Because of their role in plant protection and defense, *P*. *chlororaphis* and other *Pseudomonas* species with biopesticidal activity are being marketed for use as seed-treatment and foliar-applied biopesticides or as gene donors for GM crops (Kupferschmied et al. 2013).

### History of safe use in agriculture

#### *Pseudomonas*-based biopesticides and plant protection products

Several biopesticide products containing *Pseudomonas* species that provide protection against fungal pathogens and diseases have been developed and assessed for their safety (Table 1). For example, two strains of *P*. *syringae* (ESC-l0 and ESC-11) have been shown to control post-harvest mold contamination on certain fruits, and dry rot and silver scurf on potatoes during storage (US-EPA 1999b, 2001a, 2009b). The products developed with these strains emphasize the long history of safe use of *Pseudomonas*-based biopesticides, as they were first registered with the United States Environmental Protection Agency (US-EPA) in 1990 and 1996 (US-EPA 2017b). Over the past 30 years, several additional *Pseudomonas*-based biopesticides and plant protection products have been registered with the US-EPA or approved by the European Food Safety Authority (EFSA); this further demonstrates the long history of safety (Table 1). For example, *Pseudomonas* sp. DSMZ 13134, which is closely related to *P*. *fluorescens*, has been shown to provide protection against fungal diseases in vegetables and flowers (Buddrus-Schiemann et al. 2010; EFSA 2012), and *P*. *aureofaciens* strain Tx-1 has been shown to provide protection against fungal pathogens on golf course turf (US-EPA 1999a, 2000). *P*. *chlororaphis* strain AFS009 is being leveraged to provide protection against a range of soil-borne fungal pathogens (AgBiome 2017; US-EPA 2017a), and other strains of *P*. *chlororaphis* (strains MA 342 and 63-28) have been shown to control fungal pathogens in cereals (EFSA 2017; Johnsson et al. 1998), as well as in greenhouse ornamentals and vegetable crops (US-EPA 2001b, d). In addition to fungal protection, *Pseudomonas*-based products are used to protect plants against frost damage. For example, *P*. *syringae* is known to protect plant leaves from frost through ice nucleation (Hirano and Upper 2000), and a non-frost-forming strain of *P*. *fluorescens* (strain A506) is being used to reduce frost damage on fruit and vegetable crops (Nufarm Americas Inc. 2012; US-EPA 1992b). The same strain of *P*. *fluorescens* is also being used to suppress pathogenic bacterial growth (e.g., fire blight and russet inducing bacteria) on apple and pear crops (Nufarm Americas Inc. 2012; US-EPA 1992b), whereas *P. fluorescens* strain D7 is being used to suppress growth of certain invasive grass species (US-EPA 2014).

**Table 1:** Biopesticide products and genetically modified (GM) crops utilizing *Pseudomonas* spp. (Only naturally occurring strains of *Pseudomonas* spp. are reported) or a related species as the donor source

Species (strain)	Date first approved^a^	Product names	Use in agriculture
*Pseudomonas syringae* strains ESC-10 and ESC-11	1990 and 1996 (US-EPA)	Bio-Save^®^ 10 LP and Bio-Save^®^ 11 LP	Biopesticide—post-harvest fungicide to prevent contamination of stored fruits and potato (US-EPA 1999b, 2001a, 2009b)
*Pseudomonas fluorescens* strain A506	1992 (US-EPA)	FrostBan™	Biopesticide—provides protection from frost and suppresses bacterial pathogens (US-EPA 1992b)
*Pseudomonas aureofaciens* strain Tx-1	1999 (US-EPA)	Bio-Ject^®^ Spot-Less™	Biopesticide—foliar treatment for fungal pathogens on golf course turf (US-EPA 1999a, 2000)
*Pseudomonas chlororaphis* strain 63-28	2001(US-EPA)	AtEze™	Biopesticide—protection against fungal pathogens in greenhouse ornamentals and vegetables (US-EPA 2001b, 2001d)
*Pseudomonas sp*. strain DSMZ 13134; closely related to *P*. *fluorescens*)	2012 (EFSA)	Proradix^®^	Biopesticide—protection against fungal diseases in vegetables and flowers (Buddrus-Schiemann et al. 2010; EFSA 2012)
*Pseudomonas fluorescens* strain D7	2014 (EPA)	D7^®^	Biopesticide—suppression of certain invasive grass species (US-EPA 2014)
*Pseudomonas chlororaphis* strain MA 342	2016 (EFSA)	Cedomon^®^ and Cerall^®^	Biopesticide—protection against fungal pathogens on cereals (EFSA 2017)
*Pseudomonas chlororaphis* strain AFS009	2017 (EPA)	Howler™, Howler™ Technical, and Howler™ T&O	Biopesticide—fungicide for turf and ornamental plants (AgBiome 2017; US-EPA 2017a)
*Pseudomonas chlororaphis* strain G65	1995 (USDA)	Event 8338 tomatoes; OECD Unique Identifier CGN-89322-3	Gene donor for GM crop—*Accd* gene encodes the 1-amino-cyclopropane-1-carboxylic acid deaminase (ACCd) enzyme which reduces ethylene production and delays ripening (USDA-APHIS 1995).
*Pseudomonas fluorescens* strain A32	2013 (USDA)	Soybean event FG72; OECD Unique Identifier MST-FGØ72-2	Gene donor for GM crop—Source of recombinant DNA for GM crop; confers tolerance to isoxaflutole (IFT) herbicides when expressed in plants (USDA-APHIS 2013)
*Delftia acidovorans* (formerly classified as *Pseudomonas acidovorans*)	2014 (USDA)	OECD Unique Identifier DAS-68416-4	Gene donor for GM crop—Source of recombinant DNA for GM crop—confers tolerance to aryloxyalkanoate herbicides when expressed in plants (USDA-APHIS 2014b)
	2014 (USDA)	OECD Unique Identifier DAS-44406-6	Gene donor for GM crop—Source of recombinant DNA for GM crop—confers tolerance to aryloxyalkanoate herbicides when expressed in plants (USDA-APHIS 2014a)
	2015 (USDA)	OECD Unique Identifiers DAS-81910-7	Gene donor for GM crop—Source of recombinant DNA for GM crop—confers tolerance to aryloxyalkanoate herbicides when expressed in plants (USDA-APHIS 2015)

^a^US-EPA date indicates date first registered by the US Environmental Protection Agency (US-EPA 2017b); EFSA date indicates date first approved by the European Food Safety Authority (EFSA 2012, 2017); USDA date indicates date deregulated by US Department of Agriculture (USDA-APHIS 2017)

**Box 1:** Weight of evidence supporting the safety of *Bacillus thuringiensis* (*Bt*) as a source of insecticidal genes

Presence in the environment—ubiquitous, both in soil and on plants (Schnepf et al. 1998)
History of safe use in the field of agriculture—Bt products were initially developed as microbial pesticide sprays and have been approved for use on multiple food and feed crops (US-EPA 1998)
Phylogenetic relatedness to known human pathogens—*Bt* is not closely related to known human pathogens
Known mammalian toxic, pathogenic or allergenic potential—*Bt* is not toxic to mammals and has no known pathogenic or allergenic potential (US-EPA 1998)

As part of the registration requirements of biopesticide products, environmental and human health risk assessments are conducted prior to commercialization (US-EPA 2017c). The US-EPA concluded that these *Pseudomonas* strains are low risk, therefore these strains were granted exemptions from the requirement for a tolerance (40 CFR Parts 180.1114, 180.1145, 180.1212, 180.1304, 180.1326 and 180.1341). The human health and environmental safety of *P*. *chlororaphis* strain 63-28 and *P*. *aureofaciens* strain Tx-1 have been reviewed by the US-EPA. Both strains were determined to have no toxicity or human health concerns (US-EPA 2000, 2001d). Similarly, the human health and environmental safety of *P*. *chlororaphis* strains MA 342 and DSMZ 13134 have been reviewed by the European Commission (EC 2002; Velivelli et al. 2014) and EFSA (2012, 2017). For strain MA 342, the European Commission acknowledged that there were no signs of toxicity or pathogenicity based on a rat acute oral study, and *P. chlororaphis* is unlikely to grow at mammalian body temperature (EC 2002); EFSA recommended additional studies to finalize the risk assessment (EFSA 2017). For DSMZ 13134, EFSA concluded that this strain of *P*. *chlororaphis* is unlikely to cause toxicity or pathogenicity via oral exposure based on clinical and other experimental data (EFSA 2012).

*Pseudomonas syringae* strains ESC-10 and ESC-11 and *P. fluorescens* strain A506 were registered with the US-EPA in the early 1990s. According to the US-EPA, these strains of *P*. *syringae* pose low risk to humans or birds because they do not survive at temperatures above 32 °C, and they do not cause adverse effects in mammals when ingested, inhaled or applied topically (US-EPA 2009b). Similarly, *P*. *fluorescens* is ubiquitous in the environment, is not generally considered to be a human or animal pathogen (US-EPA 1992a) and is not expected to have adverse ecological effects on avian wildlife, aquatic organisms, non-target insects, mammalian systems or endangered species (US-EPA 1992a, 2009a).

#### *Pseudomonas* species and related species as a gene source for GM Crops

Certain *Pseudomonas* species and related species have also served as gene sources for genetically modified crops (Table 1). The GM crop products developed with these strains also emphasize the long history of safe use of *Pseudomonas* species as gene donors, as the first GM crop containing a gene from *P*. *chlororaphis* was deregulated by the United States Department of Agriculture (USDA) in 1995 (USDA-APHIS 1995, 2017). Event 8338 tomato (OECD Unique Identifier CGN-89322-3) was developed by Monsanto (Monsanto Company 1995). These GM tomatoes contain a gene from *P*. *chlororaphis* that encodes the 1-amino-cyclopropane-1-carboxylic acid deaminase (ACCd) enzyme, which has been shown to delay ripening when expressed in tomato plants by reducing ethylene production.

Similarly, in 2013 and 2014, the USDA deregulated four herbicide tolerant GM soybean and cotton varieties that were developed with genes from *P*. *fluorescens* and *Delftia acidovorans* (USDA-APHIS 2017). The gene from *P*. *fluorescens* encodes the hydroxyphenylpyruvate dioxygenase (HPPD) protein, which has been demonstrated to confer tolerance to isoxaflutole (IFT) herbicides when expressed in plants. Bayer CropScience developed herbicide tolerant soybean event FG72 (OECD Unique Identifier MST-FGØ72-2) using the *HPPD W366* gene from *P*. *fluorescens* strain A32 (USDA-APHIS 2013). The gene from *D. acidovorans* has been demonstrated to confer tolerance to aryloxyalkanoate herbicides by expression of the aryloxyalkanoate dioxygenase-12 (AAD-12) protein. Herbicide tolerance traits have been developed using the *aad*-*12* gene from *D*. *acidovorans* in soybean and cotton by Dow AgroSciences LLC [OECD Unique Identifiers DAS-44406-6; DAS-68416-4 and DAS-81910-7 (USDA-APHIS 2014a, b, 2015), respectively]. *Delftia acidovorans* was previously classified as *Pseudomonas acidovorans* and *Comamonas acidovorans*, before being reclassified recently as *Delftia* (Dow AgroSciences 2010; Tamaoka et al. 1987). The safety of both *P*. *fluorescens* and *D. acidovorans* as a gene sources for GM crops has been assessed by several regulatory authorities [for example, EFSA (2015a), FSANZ (2013), USDA-APHIS (2013) and CFIA (2013), FSANZ (2014), Health Canada (2014), USDA-APHIS (2014c), respectively]. Based on this and other evidence, GM soybean containing the gene from *P*. *fluorescens* and the GM soybean and cotton events containing the gene from *D*. *acidovorans* have been approved by several regulatory authorities globally (ISAAA 2018).

### Pathogenic, toxic or allergenic properties

As previously reviewed by Leuschner et al. (2010), regulatory authorities in the US and Europe concluded that *P*. *chlororaphis* strains used for plant protection purposes pose no health concerns for humans (EC 2002; US-EPA 2001d). Additionally, *P*. *chlororaphis* was previously reviewed by EFSA using a Qualified Presumption of Safety (QPS) Approach (EFSA 2015b), which included a thorough assessment of the species’ life history characteristics, commercial uses and safety concerns. The thorough review of *P*. *chlororaphis* safety resulted in a general consensus that it is non-pathogenic to humans and livestock because of its inability to grow and proliferate at mammalian body temperatures (EC 2002). Based on this weight of evidence, *P*. *chlororaphis* was determined to be safe for biocontrol applications (Chen et al. 2015).

While there have been a few reports where *P*. *chlororaphis* has been isolated from animals with disease or illness (for example, Hatai et al. 1975), these reports are rare and there has been no causal link to clinical illness (EC 2002; EFSA 2015b). As part of the QPS evaluation, microorganisms are considered within the context that they are “deliberately introduced in the food chain either directly or as a source of additive or food enzyme” (Leuschner et al. 2010). The QPS assessment does not consider the organism’s safety for use as a gene source for GM crops, therefore the utility of this QPS assessment is limited to applications where the organism is either used directly or as a source of additive or food enzyme in food and feed applications. The QPS assessment for *P*. *chlororaphis* noted that it may produce secondary metabolites (for example, rhamnolipids and phenazine compounds) (EFSA 2015b). However, the potential for a gene source to produce a secondary metabolite like rhamolipids or phenazine compounds does not indicate inherent risk for the GM crop. Secondary metabolites like rhamolipids or phenazine compounds are synthesized through complex biochemical pathways involving multiple genes. For example, rhamnolipids biosynthesis occurs in sequential reactions catalyzed by RhlA, RhlB and RhlC proteins [under the control of the *rhlA*, *rhlB* and *rhlC* genes, respectively (Gunther et al. 2005; Reis et al. 2011)]. Biosynthesis of phenazine compounds is controlled by *phz* genes (Dowling and O’Gara 1994). The safety of the specific gene inserted into the plant and gene products is assessed as part of the safety assessment of GM crops, and there is no evidence to suggest that the *ipd072Aa* gene from *P*. *chlororaphis* is involved in the biosynthesis of secondary metabolites like rhamnolipids or phenazine compounds.

#### Phylogenetic relatedness to known human and plant pathogens

There is currently a robust understanding of the phylogenetic relatedness within the genus *Pseudomonas* (Anzai et al. 2000; Burr et al. 2010; Garrity et al. 2005; Gomila et al. 2015; Moore et al. 2006). The *Pseudomonas* genus does contain some well-recognized plant and human pathogens, including *P*. *aeruginosa* and *P*. *syringae* (Peix et al. 2009). Therefore, the phylogenetic relatedness of pathogenic *Pseudomonas* species and other *Pseudomonas* species intended for agricultural applications should be considered before potential use. *P*. *aeruginosa* is a gram-negative, aerobic bacterium that is relatively ubiquitous in the environment and can be found in soil and water, as well as on the surface of plants. *P*. *aeruginosa* is well recognized as both a plant pathogen and an opportunistic human pathogen that can cause respiratory infection in immunocompromised patients (Sadikot et al. 2005). The pathogenicity of *P*. *aeruginosa* is thought to be related to virulence factors carried by pathogenicity islands. For example, the pathogenicity islands PAPI-1 and PAPI-2 have been linked to the virulence of *P*. *aeruginosa.* It has been confirmed that *P*. *chlororaphis* does not contain virulence factors and shares no genomic homology with these known pathogenicity islands (Chen et al. 2015). *P*. *aeruginosa* is phylogenetically distant from *P*. *chlororaphis* (Anzai et al. 2000; EC 2002; Fig. 1).

The pathogenicity of *P*. *syringae* to plants is well understood. The taxonomy of the species is separated into pathovars, each distinguishable based on the primary host plant(s) and carbon source(s) they utilize for growth (Garrity et al. 2005). The plant pathogenicity of *P*. *syringae* is based on an array of phytotoxins that produce disease symptoms. For example, *P*. *syringae* pathovar *syringae* disrupts the plasma membrane in host plants via production of syringomycins, syringopeptins and syringotoxins. *P. syringae* is phylogenetically distant from *P*. *chlororaphis* (Anzai et al. 2000; Fig. 1). Additionally, it has been confirmed that *P*. *chlororaphis* does not contain the genes that code for the biosynthesis of these or other phytotoxins or exoenzymes (cellulases, pectinases, pectin lyases) that compromise plant cell walls (EFSA 2015b).

While it is important to consider phylogenetic relatedness to known pathogens, identifying a pathogen in the same genus as a potential source donor for a GM crop does not indicate inherent risk. Many species share phylogenetic relatedness with known pathogens without being pathogenic themselves. For example, the phylogenetic relatedness of species belonging to the *Bacillus* genus has been published previously based on 16S rRNA gene sequences (see Fig. 2 in Alcaraz et al. 2010). While *Bt* shares distant phylogenetic relatedness with a few pathogens (e.g., *Bacillus anthracis*; Alcaraz et al. 2010), it has a long history of safe use as a biopesticide and as a gene source for GM crops (US-EPA 1998, 2001c). Similarly, the phylogenetic relatedness of species belonging to the *Streptomyces* genus has been published previously based on 16S rRNA gene sequences (see Fig. 1 in Kämpfer 2006). Very few species of *Streptomyces* are human, animal or plant pathogens (Kämpfer 2006). For example, *Streptomyces scabiei* is a well-known plant pathogen associated with potato scab (Zhang et al. 2016), and *Streptomyces somaliensis* is a human pathogen that causes deep tissue and bone infections (Kirby et al. 2012). Even though phylogenetically related to these pathogens, the safety of *Streptomyces viridochromogenes* as a gene source for GM crops is well established (OECD 2007).

## Conclusions

The safety assessment framework for GM crops is well established and is appropriate for assessing traits derived from non-*Bt* source organisms. One component of the safety assessment includes an evaluation of each introduced trait, including its source organism, for potential adverse pathogenic, toxic and allergenic effects. Establishing a history of safe use, and a lack of known allergenic, toxic or pathogenic properties, contributes to the weight of evidence that a gene, and its expression product (protein), derived from a source donor is safe for its intended use, when expressed in a modified crop. *Pseudomonas* species represent a potential source of genes with insecticidal properties. The *ipd072Aa* gene from *P*. *chlororaphis* encodes the IPD072Aa protein, which confers protection against certain coleopteran pests when expressed in maize plants. This paper provides an assessment of the safety of *P*. *chlororaphis* as a gene source for GM crops. Like *Bt*, *Pseudomonas* species are ubiquitous in the environment and several have been utilized in a variety of agricultural and industrial applications. Certain *Pseudomonas* species, including *P*. *chlororaphis*, have been used in biopesticide products and as a gene source for GM crops, and their safety as applied plant protection products has been previously assessed. Although *P*. *chlororaphis* is distantly related to plant and human pathogens (e.g., *P*. *aeruginosa* and *P*. *syringae)*, it is not a human, animal or plant pathogen and has no known potential to cause toxic or allergenic effects in mammals. This information supports, in part, the safety assessment of potential traits, such as IPD072Aa, derived from *P*. *chlororaphis*.
